# Co-infections in Hospitalized COVID-19 Patients- A Prospective Observational Study

**DOI:** 10.7759/cureus.30608

**Published:** 2022-10-23

**Authors:** Ramniwas Jalandra, Avinash Babu, Naveen Dutt, Nishant Kumar Chauhan, Pradeep Bhatia, Vijaya L Nag, Praveen Sharma, Deepak Kumar, Mithu Banerjee, Aditi Joshi

**Affiliations:** 1 Pulmonary Medicine, AII India Institute of Medical Sciences, Jodhpur, IND; 2 Anaesthesiology, AII India Institute of Medical Sciences, Jodhpur, IND; 3 Microbiology, AII India Institute of Medical Sciences, Jodhpur, IND; 4 Biochemistry, AII India Institute of Medical Sciences, Jodhpur, IND; 5 Internal Medicine, AII India Institute of Medical Sciences, Jodhpur, IND; 6 Allergy and Immunology, AII India Institute of Medical Sciences, Jodhpur, IND

**Keywords:** non-rebreather mask, high flow nasal cannulae, urinary tract infections, co-infections, covid-19

## Abstract

Introduction: SARS -CoV-2 was first reported in Wuhan and declared a pandemic in March 2020. Co-infections during other pandemics have been associated with severe outcomes, but data are scarce regarding co-infections in COVID-19 patients. Our study evaluated co-infections prevalence and its impact on morbidity and mortality in hospitalized COVID -19 patients.

Methods: This prospective observational study included 100 patients admitted to a high-dependency unit at a tertiary care hospital in India. Prevalence of co-infections and clinical outcome-related data were analyzed in COVID-19 patients satisfying the inclusion criteria.

Results: 14% of patients had co-infections, out of which urinary tract infection was found in 9%. Patients with co-infections had a higher mortality rate (p<0.0004). Urinary co-infection emerged as an independent risk factor for mortality (p <0.001).

Conclusion: Co-infections associated with COVID-19 infections are an essential risk factor for morbidity and mortality. Early identification and timely treatment of co-infections may help in improving clinical outcomes.

## Introduction

COVID-19 was declared a pandemic in March 2020 by the World Health Organization (WHO). As of April 14th, 2022, more than 500 million cases have been confirmed, and more than 6 million people lost their lives. Around 5-15% of patients had severe disease with the need for intensive care and mechanical ventilation [1]. Since December 2020, several SARS-CoV-2 variants have been identified. Mutations will continue as long as the covid infection spreads through a population. Despite vaccination and all protective measures, there is always a risk of severe disease and hospitalization.

Previous seasonal viral infections like seasonal or pandemic Influenza, and Middle East respiratory syndrome coronavirus (MERS-cov2) had shown a varying prevalence of bacterial/fungal respiratory co-infections [2]. Co-pathogens isolated include bacteria, fungi, and viruses. Among bacteria, the most commonly isolated organisms were Streptococcus pneumoniae, Klebsiella pneumoniae, Staphylococcus aureus, and Mycoplasma pneumoniae. Candida species were the commonly isolated fungal pathogen. Common viruses isolated were Rhinovirus/Enterovirus, Influenza B virus, Parainfluenza, Metapneumovirus, and Human retrovirus. Influenza-associated bacterial infections can lead to community-acquired pneumonia in 30% of cases [2]. A small study during the MERS outbreak showed co-infection in 10.5% of patients [3]. Co-infections during a pandemic and seasonal Influenza are associated with worse clinical outcomes [4]. So, there is a need for early diagnosis and simultaneous treatment of co-infections in COVID-19 patients. The clinical spectrum of SARS-CoV-2 infection ranges from asymptomatic to critical illness with acute respiratory distress syndrome [5]. The present study aimed to evaluate co-infections prevalence and its impact on mortality and morbidity in COVID-19 patients.

## Materials and methods

This prospective observational study was conducted at All India Institute of Medical Sciences, Jodhpur, India, including all hospitalized COVID-19 patients (> 18 years old) admitted in a COVID high dependency unit from September 2020 to January 2021. The study was conducted after approval from the institutional ethics committee. COVID-19 infection was diagnosed based on a reverse transcriptase polymerase chain reaction (RT-PCR) report of nasopharyngeal swabs, according to the Indian Council of Medical Research [6]. These patients were classified as moderate (presence of dyspnoea and or hypoxia, fever, cough with Spo2 90-93% on room air, respiratory rate more than or equal to 24/minute) or severe cases (signs of pneumonia plus one among the two - respiratory rate >30 breaths/minute and spo2 <90% in room air) of COVID-19 according to Ministry of Health and Family Welfare (MoHFW) guidelines [5].

Complete hemogram, renal function, liver function, susceptible C reactive protein, serum procalcitonin, rapid malarial antigen, dengue NS-1 antigen, dengue IgM and IgG antibody, peripheral blood smear, blood aerobic culture, urine routine microscopy, urine aerobic culture, sputum acid fast bacilli (AFB) stain and sputum aerobic culture were sent as per study protocol after enrollment at the time of admission. Chest X-ray was done as a part of lung imaging. Community-acquired co-infections were diagnosed if detected at the time of or within 48 hours of hospitalization. All patients were treated as per the standard COVID-19 protocol of MoHFW. The co-infections diagnosed were treated accordingly. Patients were followed up for 28 days or death, whichever was earlier.

Statistical analysis

The data were summarized and analyzed using the statistical package for Social Sciences (SPSS) version 23. Quantitative data were presented as mean and standard deviation (mean ± SD) if normally distributed or as the median and interquartile range (median ± IQR) if non-normally distributed. We did univariate analysis for various factors like blood culture, urine culture, sputum aerobic culture positivity, and dengue and malarial antigen as a predictor for mortality in our patients. Out of the factors with significant p-value, we did logistic regression to identify the independent risk factor for mortality. The receiver operating characteristic (ROC) curve was obtained for independent risk factors, and the area under the curve (AUC) was calculated. 

## Results

**Figure 1:**
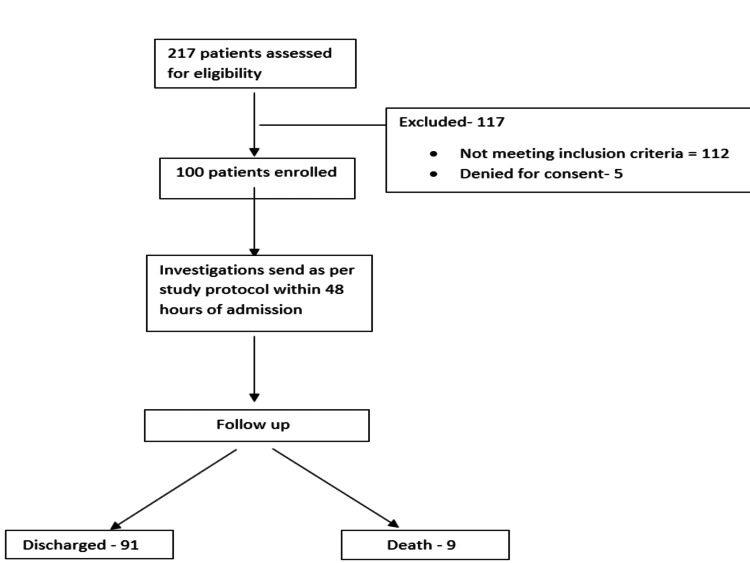
Flow chart of study

The study protocol is depicted in Figure 1. A hundred patients satisfying inclusion criteria, viz. fever (at the time of admission ± 48 hours), productive cough, shortness of breath, dysuria, abdominal pain, elevated total leukocyte count, elevated serum procalcitonin, etc., were included in the study due to end of the first wave of COVID pandemic in India till January 2021. The mean age of the patients was 58.3 ± 14.3 years. There was a male preponderance (74%) in the study group. The most common associated illnesses were diabetes mellitus (26%), primary hypertension (12%), and both in 17% of patients. The baseline characteristics of patients are given in Table 1.

**Table 1:** Baseline characteristics of patients *Out of 9 patients who received invasive mechanical ventilation during the hospital course, four were on a non-rebreather mask, three were on a high-flow nasal cannula, one was on nasal prongs, and one patient was on room air at the time of hospitalization. COPD: Chronic obstructive pulmonary disease; CRP: C-reactive protein; SGOT: Serum glutamic oxaloacetic transaminase; SGPT: Serum glutamic pyruvic transaminase

Patient characteristics (n=100)		
Age (years)		58.3± 14.3 (Mean ± SD)
Males, n (%)		74 (74%)
Females, n (%)		26 (26%)
Baseline Oxygen/ventilation support*		
Room air		8 (8%)
Nasal prongs		50 (50%)
Face mask		20 (20%)
Non-Rebreather mask		15 (15%)
High flow nasal cannula		7 (7%)
Comorbidities,		n (%)
Diabetes		26 (26%)
Hypertension		12 (12%)
Chronic kidney disease		8 (8%)
Ischemic heart disease		7 (7%)
COPD		3 (3%)
Interstitial lung disease		2 (2%)
Laboratory parameters		Median (IQR)
Highly sensitive CRP (mg/dl)		131.0 (48.7-172.0)
Procalcitonin(ng/ml)		0.16 (0.08-0.33)
Creatinine (umol/l)		88.42 (79.5-106.08)
Bilirubin(mg/dl)		0.6 (0.4-0.8)
SGOT(U/dl)		41.2 (27.6-60.9)
SGPT(U/dl)		35.3 (22.2-50.1)
Platelets(10^6^/Ul)		2.1 (1.5-2.6)
Total Leucocyte count(10^3^/Ul)		7.9 (5.3-11.4)
Hemoglobin (g/dl)		12.1(10.6-13.3)
Co-infections in patients		Total 14
Urinary co-infections		9 (64%)
	Klebsiella pneumoniae	4 (28%)
	Candida	3 (21%)
	E coli	2 (14%)
Tuberculosis		2 (14%)
Bacteremia		1 (7%)
Dengue		1(7%)
Malaria		1(7%)

Among symptoms, fever and shortness of breath were the commonest presenting complaints (96% and 92%, respectively). Cough was present in 80% of patients, whereas body aches and headaches were found in 27% and 26 % of cases, respectively. Diarrhea was seen in two patients, while one patient had hemoptysis at the time of admission. 67% of the patients had radiological evidence of bilateral diffuse consolidation, 10% had predominantly left lung consolidation, and 8% had predominantly right lung involvement. One patient had a right upper zone cavity, while another had bilateral upper lobe fibrosis on a chest x-ray. One patient had a left-sided pleural effusion. At the time of enrollment, 50% of the patients were on nasal prongs, 20% on face Masks, 15% on non-rebreather masks (NRBM), and 7% on high flow nasal cannula (HFNC), while 8% were on room air. Three out of eight patients on room air at admission needed oxygen support during illness. None of the patients were on invasive ventilation at the time of enrollment. However, nine patients had clinical worsening during illness and died after a period of invasive mechanical ventilation. Ninety-one patients were discharged from the hospital after a clinical cure.

**Figure 2:**
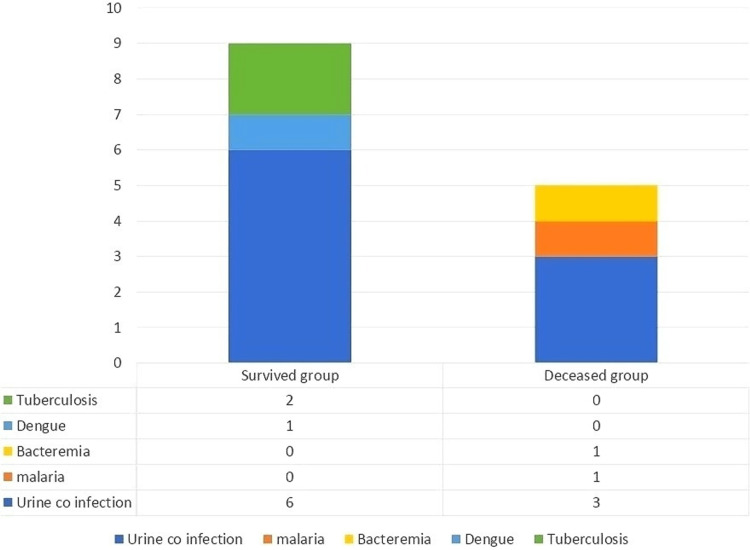
Bar diagram showing co-infections among both the groups

Out of 9 patients who received invasive mechanical ventilation during the hospital course, four were on the non-rebreather mask, three on high-flow nasal cannula, one on nasal prongs, and one on room air at the time of hospitalization. Mean serum procalcitonin and serum HsCRP were elevated in most patients (0.77+2.9ng/ml and 110.69+62.3mg/L, respectively), which were not significant predictors of mortality. Mean serum procalcitonin and serum hs-CRP in patients with co-infections were 0.264±0.3ng/ml and 93.91±69.1mg/L, respectively, which were not statistically significant. 14 out of 100 patients were diagnosed with co-infections. These were urinary tract infections, bacteremia, tuberculosis (TB), malaria, and dengue. Urine culture was positive in 9 patients (4 had Klebsiella pneumoniae, 3 had symptomatic candiduria, and 2 had E. coli). A blood culture had grown enterococcus in one patient. Tuberculosis was diagnosed in 2 patients (One patient was sputum-positive pulmonary tuberculosis, and another was tubercular pleural effusion). Both of them were discharged on anti-tubercular treatment. Dengue and cerebral malaria were diagnosed in one patient each (Figure 2).

**Figure 3:**
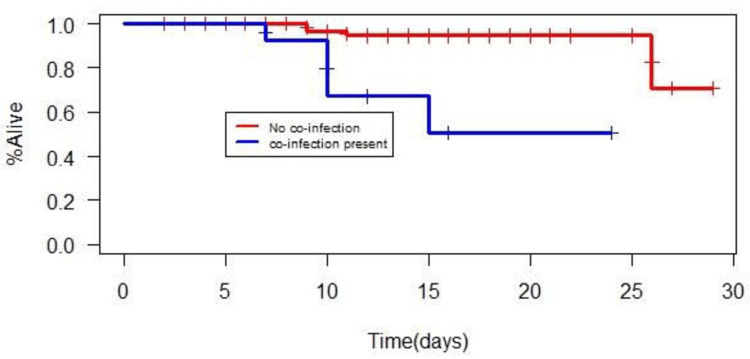
Kaplan-Meier survival analysis between patients with and without co-infections

The mean hospital stay in patients without co-infections was 11.85 ± 6.2 days. Patients with co-infections had a hospital stay of 12.69 ± 5.7 days (p-value 0.324). Compared with the group without co-infections, those with co-infections had higher mortality with a significant p-value of <0.0004. Two risk factors, urine co-infection, and malaria infection achieved significant p values on univariate analysis. Urine co-infection emerged as an independent risk factor for mortality upon logistic regression (p=0.001), showing culture positivity in 3 out of 9 (33%) patients who died (figure 3).

## Discussion

Our study focused on the diagnosis of significant co-infections (bacterial, viral, protozoan) during the COVID-19 pandemic and its impact on the clinical outcome of COVID-19 patients. The main highlights of this study are as follows: Co-infections were found in 14% of study participants, out of which urinary co-infection emerged as the independent risk factor for mortality upon logistic regression. Patients with co-infections had higher mortality than those without co-infections, which agrees with other studies [7,8]. 

To our knowledge, no prospective study regarding co-infections prevalence in hospitalized COVID-19 patients is available from the Indian subcontinent. The South-East-Asian region accounts for almost one-quarter of the world's population, and this region has a significant burden of Tuberculosis, Dengue, and Malaria. In 2019, this region accounted for 44% of the world's TB incidence and 5% of malaria cases [9]. Hence it is essential to include these infections in the diagnostic workup of community-acquired co-infections.

A retrospective study by Garcia-Vidal et al. from Spain reported 3.1% (31 of 989 patients) community-acquired co-infections in hospitalized COVID-19 patients. (2.5% bacterial and 0.6% viral) [7]. Another retrospective study from North America reported a 3.7% incidence of co-infections in hospitalized COVID-19 patients [10]. In a meta-analysis of 9 studies, Rawson et al. reported an 8% prevalence of bacterial-fungal co-infections in hospitalized COVID-19 patients. However, many studies failed to demonstrate the clinical setting [8]. It was also unclear whether the co-infections reported in the studies were community-acquired or hospital-acquired. Recent cross-sectional studies conducted in China [11-13] and the United States [14-16] with a study population ranging from 21 to 1016 patients have reported a COVID-19-associated co-infection prevalence of 3.4% to 45.0%. Most of the studies focussed on respiratory co-infections, whereas our study was elaborative with a focus on common community-acquired infections like bacterial pneumonia, tuberculosis, urinary tract infections, dengue fever, and parasitic infections like malaria, which are prevalent in our geographical region. Observational studies from the previous pandemic also reported co-infection among hospitalized patients. In 2009, during the Influenza (H1N1) pandemic, almost 1 in 4 severe cases had a bacterial co-infection with associated morbidity and mortality [17]. The most common bacteria associated with Influenza were Streptococcus pneumoniae, Staphylococcus aureus, and Streptococcus pyogenes [17,18]. Niraj et al. reported the outcomes of three pregnant women with COVID-19 who had co-infections of malaria and dengue. Fetal death has also been reported in COVID-19 patients co-infected with malaria [19]. In another retrospective study, Gupta et al. reported 13 patients of COVID-19 with active tuberculosis co-infection, out of whom 3 (23.1%) patients died. This could be due to the impact of COVID-19 on an already compromised lung [20]. 

The low prevalence of respiratory bacterial co-infection shown in our study and in other studies could result from home-based antibiotic treatment during the COVID-19 pandemic. The high incidence of urinary tract infections (9%) may be due to risk factors like diabetes, chronic kidney disease, and home-based antibiotic therapy in our study population. Our study has a few limitations. First, a well-powered study is needed to validate our findings further. Secondly, this study did not screen patients for other respiratory viral infections like H1N1 and atypical pathogens at admission. The incidence of hospital-acquired infections in these patients was not assessed, which could impact clinical outcomes.

## Conclusions

Our study concludes that in the Indian scenario, community-acquired co-infections associated with the SARS-CoV-2 pandemic may be significant risk factors for morbidity and mortality. COVID-19 and many other community-acquired infections share common presenting symptoms, so there is a need for clinical suspicion and necessary diagnostic investigations. Early identification and prompt treatment may help in improving clinical outcomes.
